# Construction and Validation of a Clinical Predictive Nomogram for Improving the Cancer Detection of Prostate Naive Biopsy Based on Chinese Multicenter Clinical Data

**DOI:** 10.3389/fonc.2021.811866

**Published:** 2022-01-21

**Authors:** Tao Tao, Changming Wang, Weiyong Liu, Lei Yuan, Qingyu Ge, Lang Zhang, Biming He, Lei Wang, Ling Wang, Caiping Xiang, Haifeng Wang, Shuqiu Chen, Jun Xiao

**Affiliations:** ^1^ Department of Urology, The First Affiliated Hospital of USTC, Division of Life Sciences and Medicine, University of Science and Technology of China, Hefei, China; ^2^ Department of Ultrasound, The First Affiliated Hospital of USTC, Division of Life Sciences and Medicine, University of Science and Technology of China, Hefei, China; ^3^ Department of Radiology, The First Affiliated Hospital of USTC, Division of Life Sciences and Medicine, University of Science and Technology of China, Hefei, China; ^4^ Department of Urology, Affiliated Zhongda Hospital of Southeast University, Nanjing, China; ^5^ Department of Urology, Shanghai East Hospital, Tongji University School of Medicine, Shanghai, China; ^6^ Department of Urology, Shanghai Changhai Hospital, Second Military Medical University, Shanghai, China

**Keywords:** prostate cancer, prostate biopsy, mpMRI, PI-RADS score, PSAD, nomogram

## Abstract

**Objectives:**

Prostate biopsy is a common approach for the diagnosis of prostate cancer (PCa) in patients with suspicious PCa. In order to increase the detection rate of prostate naive biopsy, we constructed two effective nomograms for predicting the diagnosis of PCa and clinically significant PCa (csPCa) prior to biopsy.

**Materials and Methods:**

The data of 1,428 patients who underwent prostate biopsy in three Chinese medical centers from January 2018 to June 2021 were used to conduct this retrospective study. The KD cohort, which consisted of 701 patients, was used for model construction and internal validation; the DF cohort, which consisted of 385 patients, and the ZD cohort, which consisted of 342 patients, were used for external validation. Independent predictors were selected by univariate and multivariate binary logistic regression analysis and adopted for establishing the predictive nomogram. The apparent performance of the model was evaluated *via* internal validation and geographically external validation. For assessing the clinical utility of our model, decision curve analysis was also performed.

**Results:**

The results of univariate and multivariate logistic regression analysis showed prostate-specific antigen density (PSAD) (P<0.001, OR:2.102, 95%CI:1.687-2.620) and prostate imaging-reporting and data system (PI-RADS) grade (P<0.001, OR:4.528, 95%CI:2.752-7.453) were independent predictors of PCa before biopsy. Therefore, a nomogram composed of PSAD and PI-RADS grade was constructed. Internal validation in the developed cohort showed that the nomogram had good discrimination (AUC=0.804), and the calibration curve indicated that the predicted incidence was consistent with the observed incidence of PCa; the brier score was 0.172. External validation was performed in the DF and ZD cohorts. The AUC values were 0.884 and 0.882, in the DF and ZD cohorts, respectively. Calibration curves elucidated greatly predicted the accuracy of PCa in the two validation cohorts; the brier scores were 0.129 in the DF cohort and 0.131 in the ZD cohort. Decision curve analysis showed that our model can add net benefits for patients. A separated predicted model for csPCa was also established and validated. The apparent performance of our nomogram for PCa was also assessed in three different PSA groups, and the results were as good as we expected.

**Conclusions:**

In this study, we put forward two simple and convenient clinical predictive models comprised of PSAD and PI-RADS grade with excellent reproducibility and generalizability. They provide a novel calculator for the prediction of the diagnosis of an individual patient with suspicious PCa.

## Introduction

At present, prostate cancer (PCa) is the most common malignancy of aging men worldwide. It has the highest incidence and second mortality despite the fact that the death rate is declining due to early detection of indolent cancers in western countries. Along with economic development and increased early screening tools, the incidence of PCa has also elevated rapidly in China (1, 2). Transrectal ultrasound (TRUS)-guided prostate biopsy is a standard intervention for men with suspicion of PCa (3). This operation can be performed by transrectal or transperineal approach; a few studies have demonstrated that transperineal prostate biopsy is less likely to cause infectious complications, but the cancer detection capacity of these two routes was similar when the same cores were obtained (4). With the development of imaging technology, especially the application of multiparameter magnetic resonance imaging (mpMRI), the technology of prostate biopsy has also been rapidly updated. Some new terminologies such as TRUS-guided cognitive biopsy, MRI-TRUS fusion-guided biopsy, and in-bore MRI targeted biopsy might enhance the detection rates of clinically significant PCa (csPCa) (5). But the positive rate of prostate biopsy is still miserably around 30–70%; that means almost half of the patients are overtreated and received unnecessary biopsies (3).

Apart from mpMRI, the level of serum total prostate-specific antigen (PSA) and the estimate of digital rectal examination (DRE) are two critical indicators when a clinician decides whether a patient needs prostate biopsy initially (6). DRE performed by different examiners was heterogeneous with low specificity and sensitivity (7). PSA is a serine protease, which is specifically expressed in prostate, but the elevation of PSA is not specific in PCa. It exists in benign prostate hyperplasia, prostatitis, elder men, after prostatic examinations, and sexual intercourse, and it can also be affected by taking 5α-reductase inhibitors and antiandrogen drugs (8, 9). PSA density (PSAD) is a PSA derivate, acquired by the ratio of the baseline PSA level to the prostate volume; it is useful when the PSA level is at the gray zone or patients with equivocal imaging (10). PSAD is also useful to identify patients with elevated PSA due to PCa rather than intraprostatic inflammation (11). Indeed, prostatic inflammation is a strong predictor of absence of PCa in the biopsy specimen and is associated with low-grade PCa at radical prostatectomy (12). In addition, other PSA derivates like PSA velocity, PSA rate, PSA double time, and fPSA/tPSA can also be taken into consideration when diagnosing PCa. In recent years, a number of novel molecular markers have also been explored, but their clinical value still needs more evidence before implementing them in a clinic (13, 14). Nevertheless, it is a tragedy that no available variable above can predict the diagnosis of the PCa effectively.

Actually, some risk calculators and factors have been discussed in guidelines for early screening of csPCa for asymptomatic patients with a normal DRE and a PSA value range from 2 to 10 ng/ml, but more effective tools still need to be created (15, 16). In the current study, we conducted a retrospective analysis in three regional medical centers in eastern China. A clinical predictive model was developed and validated by the data of 1,428 prostate biopsy-naive patients. We put forward two risk calculators combining PSAD and prostate imaging—reporting and data system (PI-RADS) grade, which can assess the risk probability of PCa and csPCa. The purpose of our research is to improve the detection rate of prostate cancer and reduce unnecessary prostate biopsy.

## Materials and Methods

### Study Design and Participants

A retrospective multicenter analysis was conducted in 1,428 patients from three independent regional medical centers in China. In the primary cohort, the data of 701 consecutive patients from January 2018 to June 2021 were collected in the Department of Urology at The First Affiliated Hospital of USTC and labeled as the KD cohort; nomogram development and internal validation were performed in this cohort. The data of 385 consecutive patients from January 2018 to January 2020 were collected in the Department of Urology, Affiliated Shanghai East Hospital of Tongji University and labeled as the DF cohort; the data of 342 consecutive patients from January 2018 to March 2020 were collected in the Department of Urology at Affiliated Zhongda Hospital of Southeast University and labeled as the ZD cohort. External validation was performed in these two cohorts. Ethical approval was received from the respective institutional ethics committee, and a signed informed consent was required for every participant before the biopsy.

### Baseline Data Collection and Processing

The baseline clinicopathologic information including age, BMI (body mass index) (kg/m^2^), serum PSA (ng/ml), mpMRI-based prostate volume (maximum anteroposterior diameter × maximum transverse diameter × maximum longitudinal diameter × 0.52, ml) (17), and PI-RADS score was collected from medical records. PSAD was defined as the ratio of the total PSA value to the prostate volume. A set of inclusion and exclusion criteria was formulated for screening the eligible patients in the three medical centers. ① Only the prostate naive biopsy was considered in this study. ② The enrolled cases must have complete baseline clinicopathologic information; patients with any missing value will be discarded immediately. ③ Laboratory data must be collected within one week before biopsy. ④ Patients with a history of other malignancies or a family history of PCa were excluded. ⑤ None received anti-androgen therapy or took 5α-reductase inhibitors before biopsy. ⑥ Patients with extreme serum PSA values (PSA≥100 ng/ml or PSA<4 ng/ml) were eliminated. The results of the digital rectal examination (DRE) and the fPSA-to-tPSA ratio were not analyzed because the proportion of missing data was >30% in the KD cohort.

### MRI Image Acquisition and Interpretation

Patients enrolled in our study underwent a 3.0T MRI scanner with an external 6-channel body array coil (Trio Tim, Siemens Healthineers, Erlangen, Germany) in the KD cohort, a 3.0T system (Magnetom Skyra, Siemens Medical Solutions, Erlangen, Germany) with an 18-channel phased-array coil in the DF cohort, and a 3.0T scanner with an external 8-channel body array coil (PHILIPS, MR Systems Ingenia) in the ZD cohort. The images that required procedure were operated by experienced and professional radiologists within 2 months before biopsy. In order to maintain consistency and authenticity of the data, MRI performed not in the corresponding hospital of the three cohorts or if only a writing report was provided in the medical records was not acceptable. At least axial T2-weighted imaging (T2WI) and diffusion-weighted imaging (DWI) with a quantitative apparent diffusion coefficient (ADC) picture (*b* values were 0, 800, and 1,400 s/mm^2^ in the KD cohort; 0, 800, 1,000, and 2,000 s/mm^2^ in the DF cohort; and 0, 1,000, and 2,000 s/mm^2^ in the ZD cohort) were obtained for the image interpretation according to the PI-RADS v2.1 (18).

Two radiologists who were blinded to histopathological results with more than 3 years of experience in prostate imaging in each medical center were invited to review the images. They first interpreted the MRI images independently and discussed the inconsistent results together subsequently. Finally, an official MRI report with a PIRADS score from 1 to 5 on the basis of PI-RADS v2.1 was obtained for every involved patient. In this study, we divided the PI-RADS scores into three grades: Grade 1 (PI-RADS 1 and 2) represented very low or low probability of PCa; grade 2 (PI-RADS 3) represented intermediate probability of PCa; and grade 3 (PI-RADS 4 and 5) represented high or very high probability of PCa (19).

### Prostate Biopsy and Pathology

For all the patients who entered into our study, they underwent TRUS (biplane imaging scan)-guided prostate biopsy operated by professional urologists. A systematic 12-core biopsy was performed first. Next the 0–6 cores of cognitive fusion target biopsy of the suspicious lesions in mpMRI or ultrasound were also performed in all the three medical centers. So, the patient who only underwent target biopsy would be excluded. The primary endpoint of this study is cancer detection of the prostate biopsy. Positive result is defined as PCa with gleason score≥6 (3 + 3). The second endpoint was the detection of csPCa (gleason score≥7). Any other diagnosis like normal prostate gland tissue, benign prostatic hyperplasia, or prostate tissue with inflammation was defined as a negative result.

### Model Construction, Validation, and Statistical Analysis

The descriptive statistics means ± standard deviation, interquartile range (IQR), range, number, and proportions were used to depict the baseline characteristics of the patients in primary and validation cohorts. Data in the KD cohort were used for model development. The univariate binary logistic regression analysis method was used to evaluate different variables and calculate the odds ratio (OR) and 95% confidence interval (95%CI). Next, variables with P value<0.05 were entered into the stepwise (forward: conditional) multivariate logistic regression analysis model; variables with P value<0.05 in multivariate analysis were adopted for the establishment of the nomograms. Then, the apparent performance of our nomograms was evaluated *via* internal validation in the KD cohort by the bootstrap (500 resamples) method and geographically external validation in the DF and ZD cohorts. Discrimination and calibration were assessed for model validation, respectively (20). Discrimination was measured by C-statistics, which is equal to the area under the curve (AUC) calculated by plotting the receiver operating characteristic (ROC) curve (21); calibration was measured by drawing calibration curves, and the brier score was calculated by the equation (Y-*p*)^2^, where Y is the actual observed outcome of the dependent variable, and *p* represents the predicted probability given by our nomogram (22). Statistical analysis was operated by SPSS version 25.0 and R version 4.1.1; statistical significance was considered when P<0.05.

### Decision Curve Analysis

Apart from providing a quantitative nomogram for urologists to predict the probability of prostate cancer after biopsy, a decision curve analysis (DCA) was also constructed to estimate the clinical utility of the nomograms. The clinical net benefits of our model at different threshold probabilities were quantified (23). This was accomplished by using the R software.

## Results

### Demographic and Clinicopathologic Characteristics of the Patients

All the clinical characteristics of the developed and validated cohorts are summarized in **Table 1**. As for patient selection, it must comply with the aforementioned inclusion and exclusion criteria strictly. Ultimately, 701 patients met the criteria in the KD cohort, 385 patients met the criteria in the DF cohort, and 342 patients were enrolled in the ZD cohort. The average age, BMI, PSA, and PSAD were 68.76 ± 8.97 (years), 23.32 ± 2.55 (kg/m^2^), 20.21 ± 17.63 (ng/ml), and 0.62 ± 0.78 in the KD cohort; 66.36 ± 8.35 (years), 23.90 ± 3.11 (kg/m^2^), 12.76 ± 10.42 (ng/ml), and 0.41 ± 0.58 in the DF cohort; and 68.65 ± 8.82 (years), 24.39 ± 3.01 (kg/m^2^), 16.74 ± 15.67 (ng/ml), and 0.41 ± 0.51 in the ZD cohort, respectively. The proportion in the different PSA group and the PI-RADS score grade of the patients in the three cohorts is also displayed in **Table 1**. The positive rate of prostate biopsy was 43.94%, 51.17%, and 40.06%, and the percentage of csPCa was 34.09%, 42.08%, and 34.21% in the KD, DF, and ZD cohorts, respectively.

**Table 1:** Demographic characteristics of the patients in development cohort and validation cohorts.

Clinicopathological parameters		KD cohort (n = 701)	DF cohort (n = 385)	ZD cohort (n = 342)
Age (years)		68.76 ± 8.97	66.36 ± 8.35	68.65 ± 8.82
	IQR&Range	12.00 (33.00-90.00)	11.00 (44.00-89.00)	12.25 (34.00-91.00)
BMI (kg/m2)		23.32 ± 2.55	23.90 ± 3.11	24.39 ± 3.01
	IQR&Range	3.65 (14.90-33.50)	3.58 (14.83-32.98)	3.71 (16.56-37.50)
PSA (ng/ml)		20.21 ± 17.63	12.76 ± 10.42	16.74 ± 15.67
	IQR&Range	13.00 (4.28-98.56)	7.47 (4.00-89.72)	11.40 (4.06-99.32)
Group 1 n (%)	4≤PSA<10	204 (29.10%)	195 (50.65%)	143 (41.81%)
Group 2 n (%)	10≤PSA<20	281 (40.09%)	143 (37.14%)	120 (35.09%)
Group 3 n (%)	20≤PSA<100	216 (30.81%)	47 (12.21%)	79 (23.10%)
PSAD		0.62 ± 0.78	0.41 ± 0.58	0.41 ± 0.51
	IQR&Range	0.48 (0.03-5.64)	0.29 (0.05-6.17)	0.32 (0.04-4.95)
PI-RADS score				
Grade 1 n (%)	1-2	346 (49.36%)	141 (36.62%)	104 (30.41%)
Grade 2 n (%)	3	91 (12.98%)	72 (18.70%)	98 (28.65%)
Grade 3 n (%)	4-5	264 (37.66%)	172 (44.68%)	140 (40.94%)
Pathology				
n (%)	positive	308 (43.94%)	197 (51.17%)	137 (40.06%)
	csPCa	239 (34.09%)	162 (42.08%)	117 (34.21%)
n (%)	negative	393 (56.06%)	188 (48.83%)	205 (59.94%)

IQR, interquartile range; BMI, body mass index; PSA, prostate-specific antigen; PSAD, prostate-specific antigen density; PI-RADS, prostate imaging-reporting and data system; csPCa, clinically significant prostate cancer.

### Variables Screening, Nomogram Development, and Internal Validation

**Figure 1:**
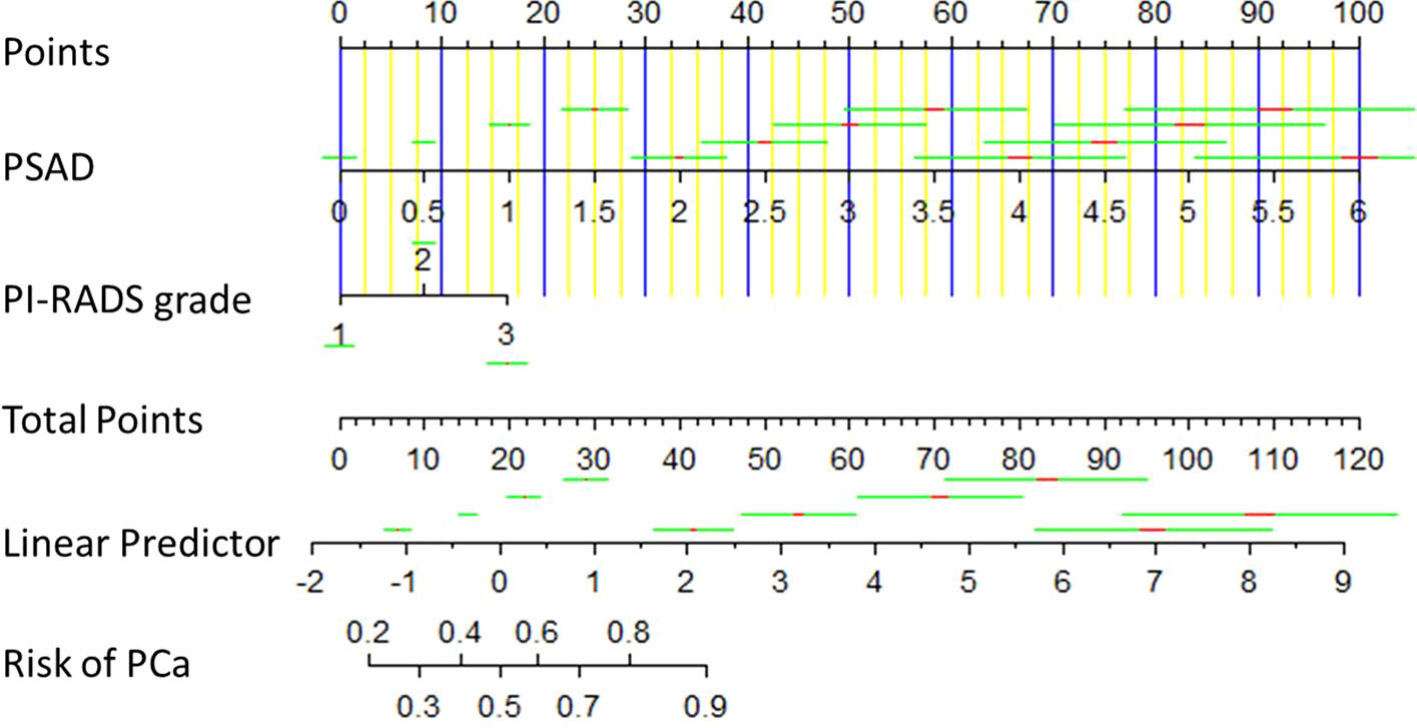
Diagnostic nomogram for predicting the outcome of prostate biopsy. It was established by the development cohort. A total point was calculated by combining PSAD and PI-RADS grade, which parallels to a risk value of PCa.

**Figure 2:**
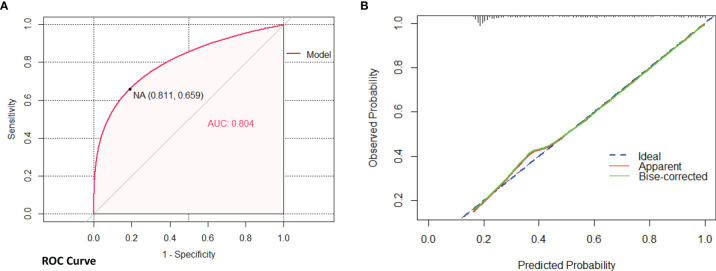
Internal validation of nomogram (PCa) in the KD cohort by bootstrap method (500 resamples). **(A)** Discrimination of the nomogram was evaluated by the ROC curve; AUC=0.804 which is equal to a c-statistic. **(B)** Calibration curves illuminate the agreement between the predicted risks of PCa and the observed incidence of PCa. The blue dotted line represents an ideal flawless model.

Binary logistic regression analysis was used for sifting the predictors of PCa in the derivation cohort (the KD cohort). The results of univariate analysis showed that age (P<0.001, OR:1.063, 95%CI:1.043–1.083), BMI (P<0.05, OR:1.071, 95%CI:1.010–1.136), PSA (P<0.01, OR:1.012, 95%CI:1.004–1.021), PSAD (P<0.001, OR:10.906, 95%CI:6.567–18.112), and PI-RADS grade (P<0.001, OR:3.278, 95%CI:2.714–3.959) were significantly associated with the outcome of prostate biopsy. We put these variables into multivariate analysis subsequently, the result indicated that PSAD (P<0.001, OR:2.102, 95%CI:1.687–2.620) and PI-RADS grade (P<0.001, OR:4.528, 95%CI:2.752–7.453) were independent predictors for PCa (**Table 2**). Therefore, a predictive model containing PSAD and PI-RADS grade was established, and a nomogram was also constructed based on our model (**Figure 1**). Internal validation of the KD cohort showed that the predictive nomogram has good discrimination (AUC=0.804) (**Figure 2A**), and the calibration curve indicated that the predicted incidence of PCa was consistent with the observed incidence in the KD cohort (**Figure 2B**); the brier score was 0.172. These results indicated that the modeling process has great reproducibility. A separated predicted nomogram of csPCa was constructed with the same processes; the final variables in the model were also PSAD (P<0.001, OR:2.480, 95%CI:1.947–3.157) and PI-RADS grade (P<0.001, OR:4.769, 95%CI:3.013–7.548) (**Table S1**). Then, the nomogram (**Figure S1**), the ROC curve (AUC=0.848) (**Figure S2A**), the calibration curve (**Figure S2B**), and the brier score (0.138) for internal validation were also received.

**Table 2:** Univariate and multivariate analysis for screening the predictors of outcomes (PCa) of prostatic biopsy.

Parameters	Univariate model			Multivariate model			
	OR	95%CI	P	B	OR	95%CI	P
Age (years)	1.063	1.043-1.083	<0.001				
BMI (kg/m^2^)	1.071	1.010-1.136	0.023				
PSA (ng/ml)	1.012	1.004-1.021	0.006				
PSAD	10.906	6.567-18.112	<0.001	0.743	2.102	1.687-2.620	<0.001
PI-RADS grade	3.278	2.714-3.959	<0.001	1.510	4.528	2.752-7.453	<0.001

PCa, prostate cancer; BMI, body Mass index; PSA, prostate-specific antigen; PSAD, prostate-specific antigen density; PI-RADS, prostate imaging-reporting and data system; OR, odds ratio; CI, confidence interval.

### External Validation and Clinical Application

**Figure 3:**
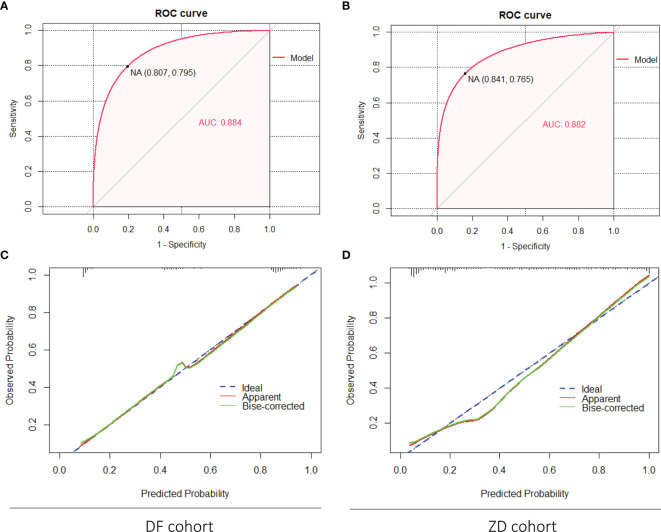
External validation of the nomogram (PCa) in the DF cohort and the ZD cohort. **(A, B)** Discrimination of the nomogram was evaluated by the ROC curve; AUC was 0.884 in the DF cohort and 0.882 in the ZD cohort. Calibration curves of the DF cohort **(C)** and the ZD cohort **(D)** illuminate the great agreement between the predicted risks of PCa and the observed incidence of PCa. The blue dotted line represents an ideal flawless model.

External validation was implemented in the DF and ZD cohorts, respectively. Similarly, discrimination and calibration were estimated by the AUC of ROC curves and calibration plots; the brier score was also calculated. For the nomogram of PCa, acceptable results were obtained in both two validation cohorts; the AUC value was 0.884 in the DF cohort and 0.882 in the ZD cohort (**Figures 3A, B**). Calibration plots elucidated greatly predicted the accuracy of PCa in two validation cohorts (**Figures 3C, D** **)**; the brier score was 0.129 in the DF cohort and 0.131 in the ZD cohort. These results indicated a good agreement between the predicted risk of PCa and observed outcomes. Meanwhile, similar results obtained for the nomogram of csPCa, AUC values (0.859 in DF cohort and 0.892 in ZD cohort) (**Figures S3A, B**), calibration plots (**Figures S3C, D**), and the brier score (0.149 in DF cohort and 0.119 in ZD cohort) were displayed. All these data demonstrated that our models possessed excellent generalizability.

### Decision Curve Analysis

**Figure 4:**
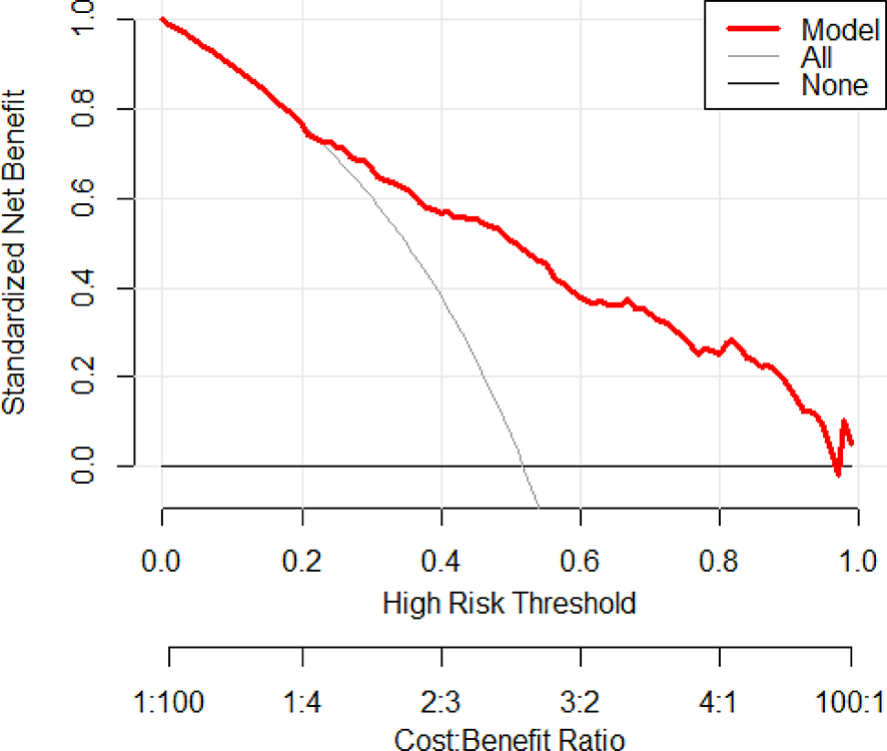
Decision curve analysis was exhibited to estimate the clinical usefulness of the nomogram (PCa). The quantified net benefits can be measured at different threshold probabilities. The y-axis denotes the standardized net benefit, and the x-axis denotes the threshold probabilities. The red line represents our nomogram, the gray line represents the condition that all patients have PCa, and the black line represents the condition that none have PCa.

For evaluating the clinical usefulness of our nomograms, the decision curve analysis (DCA) was exhibited (**Figure 4** and **Figure S4**). The DCA curves showed that along with the increase in the probability threshold, making the decision of whether to undergo prostate biopsy while referring to our predicted models can add net benefit compared to intervening all patients or intervening none.

### Internal and External Validation in Different PSA Groups

As we all know, PSA is the most commonly used indicator for early screening of PCa in men>50 years old with life expectancy >15 years (15). Although multivariate logistic analysis revealed that PSA was not an independent factor of PCa in the current study, the PSA level is still the most important serum test when urologists decide whether a man should undergo prostate biopsy. Therefore, we divided the patients into 3 groups according to the different PSA level (4≤PSA<10 defined as group 1, 10≤PSA<20 defined as group 2, and 20≤PSA<100 defined as group 3) in the development cohort and two validation cohorts and put our model into these groups for validation. As we expect, our predictive model presented encouraging performance.

For internal validation of the KD cohort, the AUC of ROC was 0.689 in group 1, 0.791 in group 2, and 0.905 in group 3 (**Figures S5A–C**); the brier score was 0.204 in group 1, 0.179 in group 2, and 0.121 in group 3 (**Table 3**). For external validation of the DF cohort, the AUC of ROC was 0.867, 0.909, and 0.885 in groups 1, 2, and 3, respectively (**Figures S6A–C**); the brier score was 0.133, 0.106, and 0.083 in groups 1, 2, and 3, respectively (**Table 3**). For external validation of the ZD cohort, the AUC of ROC was 0.769, 0.906, and 0.914 in groups 1, 2, and 3, respectively (**Figures S7A–C**); the brier score was 0.145 in group 1, 0.118 in group 2, and 0.098 in group 3 (**Table 3**). Calibration curves received acceptable results as well (**Figures S5**–**S7D–F**). All these results proved that our nomogram has excellent predictive ability in different PSA groups. That means the application of this nomogram can provide reliable evidence in clinical decision-making, especially when the PSA is 4–10 ng/ml, which is called the gray zone by urologists.

**Table 3:** The results of internal and external validation of the nomogram in different PSA group.

Cohorts	Parameters	All patients	4≤PSA<10	10≤PSA<20	20≤PSA<100
			Group 1	Group 2	Group 3
KD cohort					
(Internal validation)	AUC	0.804	0.689	0.791	0.905
	Brier score	0.172	0.204	0.179	0.121
DF cohort					
(External validation 1)	AUC	0.884	0.867	0.909	0.885
	Brier score	0.129	0.133	0.106	0.083
ZD cohort					
(External validation 2)	AUC	0.882	0.769	0.906	0.914
	Brier score	0.131	0.145	0.118	0.098

PSA, prostate-specific antigen; AUC, area under the curve.

## Discussion

mpMRI is increasingly performed in clinics for the diagnosis of PCa. There are several high-quality studies specifically assessing the efficiency and availability of mpMRI in recent years (24–29). In the PROMIS study (26), they found that mpMRI can be regarded as a triage test before prostate naive biopsy and help a quarter of patients avoid an unnecessary biopsy. For the diagnosis of csPCa in men with PSA up to 15 ng/ml, the sensitivity and specificity of mpMRI and TRUS-biopsy were 93% and 41%, and 48% and 96%, respectively. In the PRECISION study (27), the detection rate of csPCa can increase by 12% by MRI-targeted biopsy (38%) compared to standard biopsy (26%), and less clinically insignificant PCa was detected in the MRI-targeted biopsy group synchronously. But in the MRI-FIRST study (28), they discovered that the positive rate of PCa with MRI-targeted biopsy was adjacent to systematic biopsy. A better outcome can be achieved by combining systematic biopsy with targeted biopsy. In the Trio study (29), they confirmed that MRI-targeted biopsy had lower ability in grade group 1 PCa but showed an outstanding cancer detection rate in higher-grade PCa. Moreover, 8% csPCa would be missed if only MRI-targeted biopsy is performed. A study by Rapisarda et al. (30) also confirmed that mpMRI could improve the diagnostic accuracy of PCa; a combined strategy of fusion targeted and systematic biopsy could reach high concordance rates with histologic result. Collectively, mpMRI has brilliant performance in the detection of csPCa, but it cannot in the case of systematic biopsy completely. In addition, guidelines have recommended mpMRI in patients with suspicion of PCa prior to biopsy (15, 31). It is worth noting that mpMRI cannot be performed as an initial screening tool because the low specificity may lead to false-positive findings and unnecessary biopsies (32). PI-RADS is an interpretation of mpMRI, which can give a quantitative score range from 1 to 5, in which a high score means high probability of PCa. PI-RADS v2.1 is the latest version with enhanced interreader variability and easier PI-RADS assessment procedure compared to PI-RADS v2 (18, 33).

Actually, some predictive models have been set up before for the detection of PCa. Two risk calculators of western patients had been well developed; they are The European Randomized Study of Screening for PCa Risk Calculator and the Prostate Cancer Prevention Trial Risk Calculator. However, it will result in approximately 20% increase in predicted probabilities for PCa in Chinese population on account of the differences of race and region (34, 35). A number of nomograms for the detection of PCa based on Chinese population have been constructed as well recently. Chen et al. (36) constructed the Chinese Prostate Cancer Consortium Risk Calculator (CPCC-RC) based on PSA, age, prostate volume, fPSA-to-tPSA rate, and DRE for forecasting the initial prostate biopsy. This is the largest research of China to date, but the information of mpMRI was not estimated. A clinical nomogram including mpMRI like that of Fang et al. (19) showed that the model that contained mpMRI exhibited higher sensitivity and specificity for the detection of PCa. Niu et al. (37) developed an outperforming model composed of the PI-RADS v2 score and adjusted PSAD. Li et al. (38) displayed three radiomics prediction models for improving the diagnosis of csPCa on biparametric MRI. Studies including our previous research also verified the prominent diagnostic efficiency of PI-RADS v2. But all these studies were executed in a single center with an insufficient sample size (39–42). To improve the detection of PCa and reduce the needless biopsy procedures, a more accurate diagnostic nomogram and better predictive model is still warranted (43).

In the current study, we performed a retrospective study in three Chinese medical centers. Univariate and multivariate logistic regression analysis revealed that PSAD (P<0.001, OR:2.102, 95%CI:1.687–2.062) and PI-RADS grade (P<0.001, OR:4.528, 95%CI:2.752–7.453) were independent predictors for the diagnosis of PCa and used for establishing the predictive model and the nomogram. Then, model validation in the KD cohort (internal validation) and the DF and ZD cohorts (external validation) was performed. The AUC (0.804) indicated good discrimination; the calibration curve and brier score (0.172) also represent eminent calibration in internal validation. In external validation, AUC was 0.884 in the DF cohort and 0.882 in the ZD cohort, calibration curves elucidated greatly predicted the accuracy of PCa, and the brier score was 0.129 and 0.131 in the DF and ZD cohorts, respectively. Decision curve analysis (DCA) manifested that our nomogram can obviously add the net benefit when forecasting the diagnosis of PCa. Finally, we verified our model in three different PSA groups; acceptable results have also been obtained. In addition to our research, the latest two studies have also demonstrated that the utility of PSAD in addition to MRI PIRADS score can assist in the individualized decision-making process prior to prostate biopsy (44, 45).

Our study also has several limitations. First, our study was enforced in three third-grade class A hospitals in China; it may not be reproducible in less experienced medical centers or other countries. Then, clinicopathological parameters like the results of DRE and the fPSA-to-tPSA rate were not included due to the irretrievably missing value. Third, although the model has excellent performance in our research, it may not be applicable to all the patients, and the dilemma is still going to happen. Combining with different tools such as SelectMDx, 4Kscore can also be considered under special circumstances (46, 47). Finally, although we carried out a set of inclusive and exclusive criteria during the data collection, bias could not be completely avoided. Such as the protocol of biopsy and mpMRI interpretation by different clinicians, inter-institutional outcomes could not be well evaded. In practice, this phenomenon is inevitable because each hospital is independent; it also means our model has good generalizability. Furthermore, a study by Ugo G et al. (48) discovered that the diagnostic accuracy of mpMRI in PCa is not different between races; that means our nomogram can also be applied in other populations.

## Conclusions

We established two simple and convenient clinical predictive nomograms comprised of PSAD and PI-RADS grade with excellent reproducibility and generalizability. They are novel risk calculators for the prediction of the diagnosis of PCa and csPCa in China. But prospective validation and update remain warranted.

## Data Availability Statement

The raw data supporting the conclusions of this article will be made available by the authors, without undue reservation.

## Ethics Statement

The studies involving human participants were reviewed and approved by the Ethics Committee of USTC, the Ethics Committee of Southeast University, and the Ethics Committee of Tongji University. The patients/participants provided their written informed consent to participate in this study.

## Author Contributions

Conception, design, and manuscript reviewing: JX, SC, and HW. Data analysis and manuscript drafting: TT, CW, and WL. Data collection and sorting: LY, QG, LZ, BH, LeW, LiW, and CX. All authors contributed to the article and approved the submitted version.

## Funding

This study was supported by the National Natural Science Foundation of China (No. 81702540 and No. 82072807), the Natural Science Foundation of Anhui Province (No. 2108085MH293), and Key Research and Development Project of Anhui Province (No. 202004J07020022).

## Conflict of Interest

The authors declare that the research was conducted in the absence of any commercial or financial relationships that could be construed as a potential conflict of interest.

## Publisher’s Note

All claims expressed in this article are solely those of the authors and do not necessarily represent those of their affiliated organizations, or those of the publisher, the editors and the reviewers. Any product that may be evaluated in this article, or claim that may be made by its manufacturer, is not guaranteed or endorsed by the publisher.

## Supplementary Material

Supplementary Figure 1 Diagnostic nomogram for predicting clinically significant prostate cancer(csPCa) of prostate biopsy. It was established by the development cohort. A total point was calculated by combining PSAD and PI-RADS grade which parallels to a risk value of csPCa.

Supplementary Figure 2 Internal validation of the nomogram (csPCa) in the KD cohort by bootstrap method (500 resamples). (A) Discrimination of the nomogram was evaluated by AUC (0.848). (B) Calibration curves illuminate the agreement between the predicted risks of csPCa and the observed incidence of csPCa. The blue dotted line represents an ideal flawless model.

Supplementary Figure 3 External validation of the nomogram (csPCa) in the DF cohort and the ZD cohort. (A, B) Discrimination of the nomogram was evaluated by AUC; it was 0.859 in the DF cohort and 0.892 in the ZD cohort. Calibration curves of the DF cohort (C) and the ZD cohort (D) illuminate the great agreement between the predicted risks of csPCa and the observed incidence of csPCa. The blue dotted line represents an ideal flawless model.

Supplementary Figure 4 Decision curve analysis was exhibited to estimate the clinical usefulness of the nomogram (csPCa). The quantified net benefits can be measured at different threshold probabilities. The y-axis denotes the standardized net benefit, and the x-axis denotes the threshold probabilities. The red line represents our nomogram, the gray line represents the condition that all patients have csPCa, and the black line represents the condition that none have csPCa.

Supplementary Figure 5 Internal validation of the nomogram in the KD cohort for three PSA groups. (A, B, and C) ROC curve of the three groups for assessing the discrimination. (D, E, and F) Calibration plots of the three groups for assessing the calibration.

Supplementary Figure 6 External validation of the nomogram in the DF cohort for three PSA groups. (A, B, and C) The ROC curve of the three groups for assessing the discrimination. (D, E, and F) Calibration plots of the three groups for assessing the calibration.

Supplementary Figure 7 External validation of the nomogram in the ZD cohort for three PSA groups. (A, B, and C) The ROC curve of the three groups for assessing the discrimination. (D, E, and F) Calibration plots of the three groups for assessing the calibration.

Supplementary Table 1 Univariate and multivariate analysis for screening the predictors of outcomes (csPCa) of prostatic biopsy. csPCa, clinically significant prostate cancer; BMI, body mass index; PSA, prostate-specific antigen; PSAD, prostate-specific antigen density; PI-RADS, prostate imaging-reporting and data system; OR, odds ratio; CI, confidence interval.
