# Supplementary figures and images for: Construction and Validation of a Clinical Predictive Nomogram for Improving the Cancer Detection of Prostate Naive Biopsy Based on Chinese Multicenter Clinical Data
Source: Front Oncol. 2022 Jan 21;11:811866. doi: 10.3389/fonc.2021.811866 (PMC8814531; doi:10.3389/fonc.2021.811866)

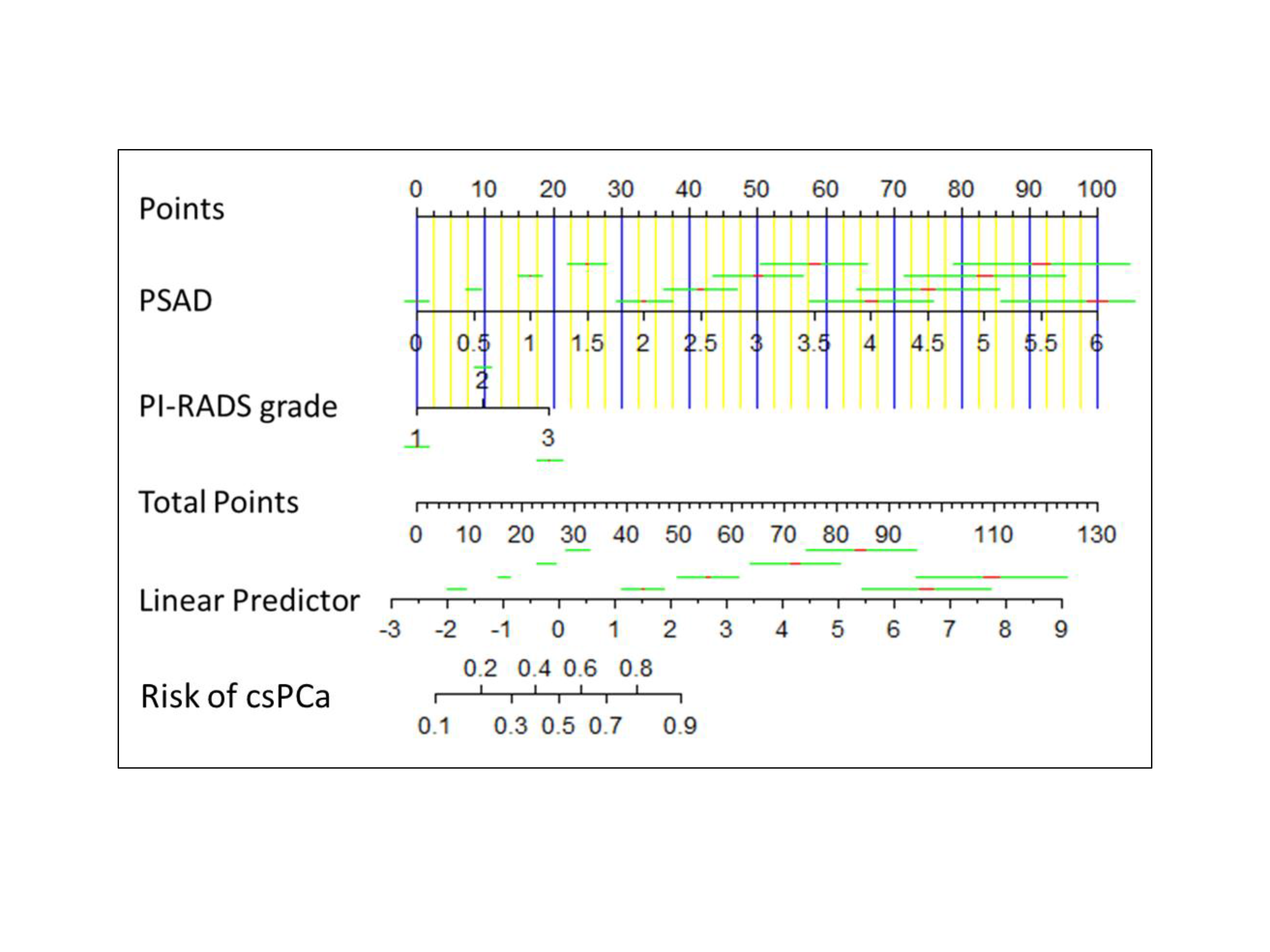

Supplement: Supplementary Figure 1 — Diagnostic nomogram for predicting clinically significant prostate cancer(csPCa) of prostate biopsy. It was established by the development cohort. A total point was calculated by combining PSAD and PI-RADS grade which parallels to a risk value of csPCa. [file Image_1.tif]

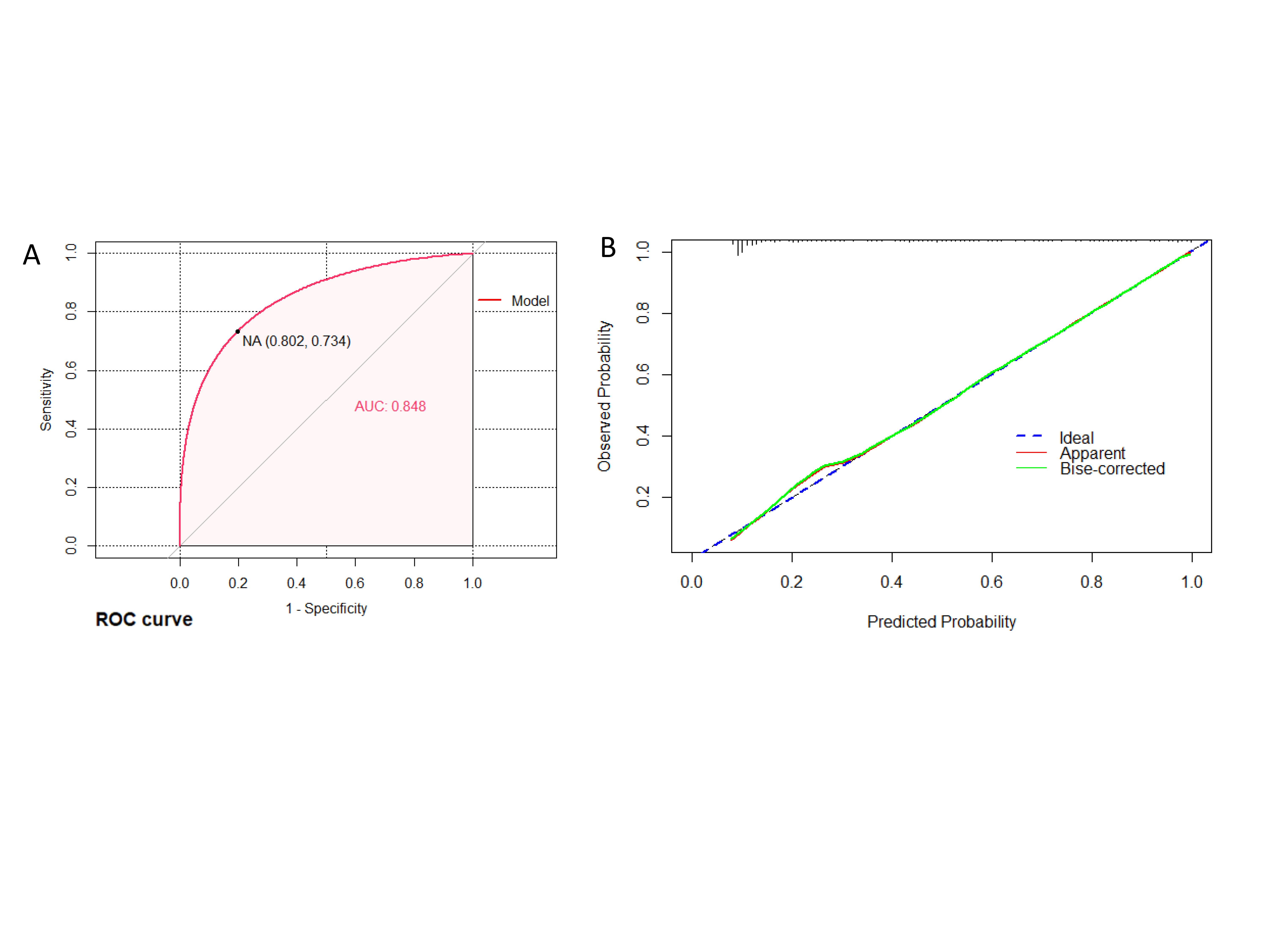

Supplement: Supplementary Figure 2 — Internal validation of the nomogram (csPCa) in the KD cohort by bootstrap method (500 resamples). (A) Discrimination of the nomogram was evaluated by AUC (0.848). (B) Calibration curves illuminate the agreement between the predicted risks of csPCa and the observed incidence of csPCa. The blue dotted line represents an ideal flawless model. [file Image_2.tif]

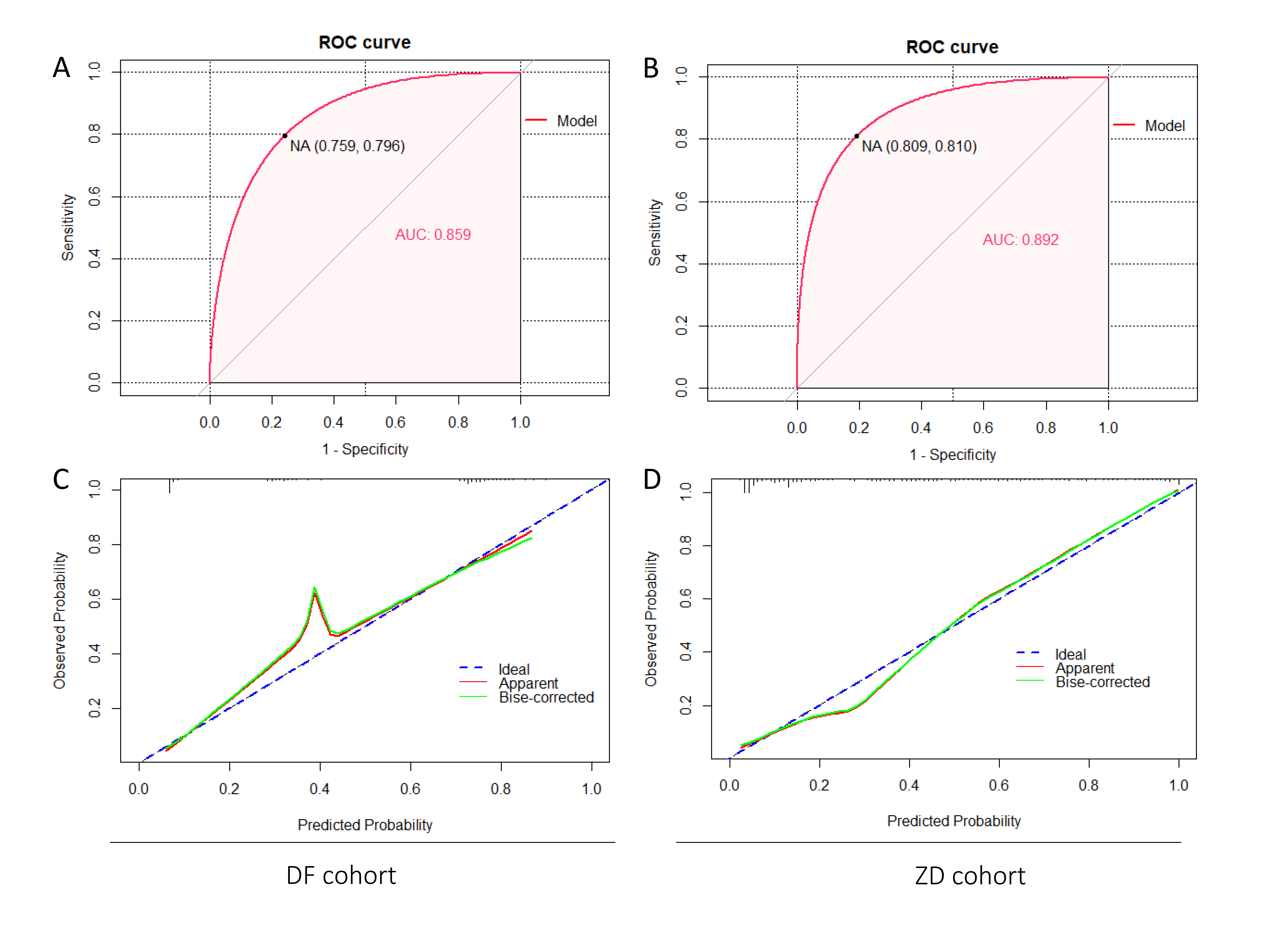

Supplement: Supplementary Figure 3 — External validation of the nomogram (csPCa) in the DF cohort and the ZD cohort. (A, B) Discrimination of the nomogram was evaluated by AUC; it was 0.859 in the DF cohort and 0.892 in the ZD cohort. Calibration curves of the DF cohort (C) and the ZD cohort (D) illuminate the great agreement between the predicted risks of csPCa and the observed incidence of csPCa. The blue dotted line represents an ideal flawless model. [file Image_3.tif]

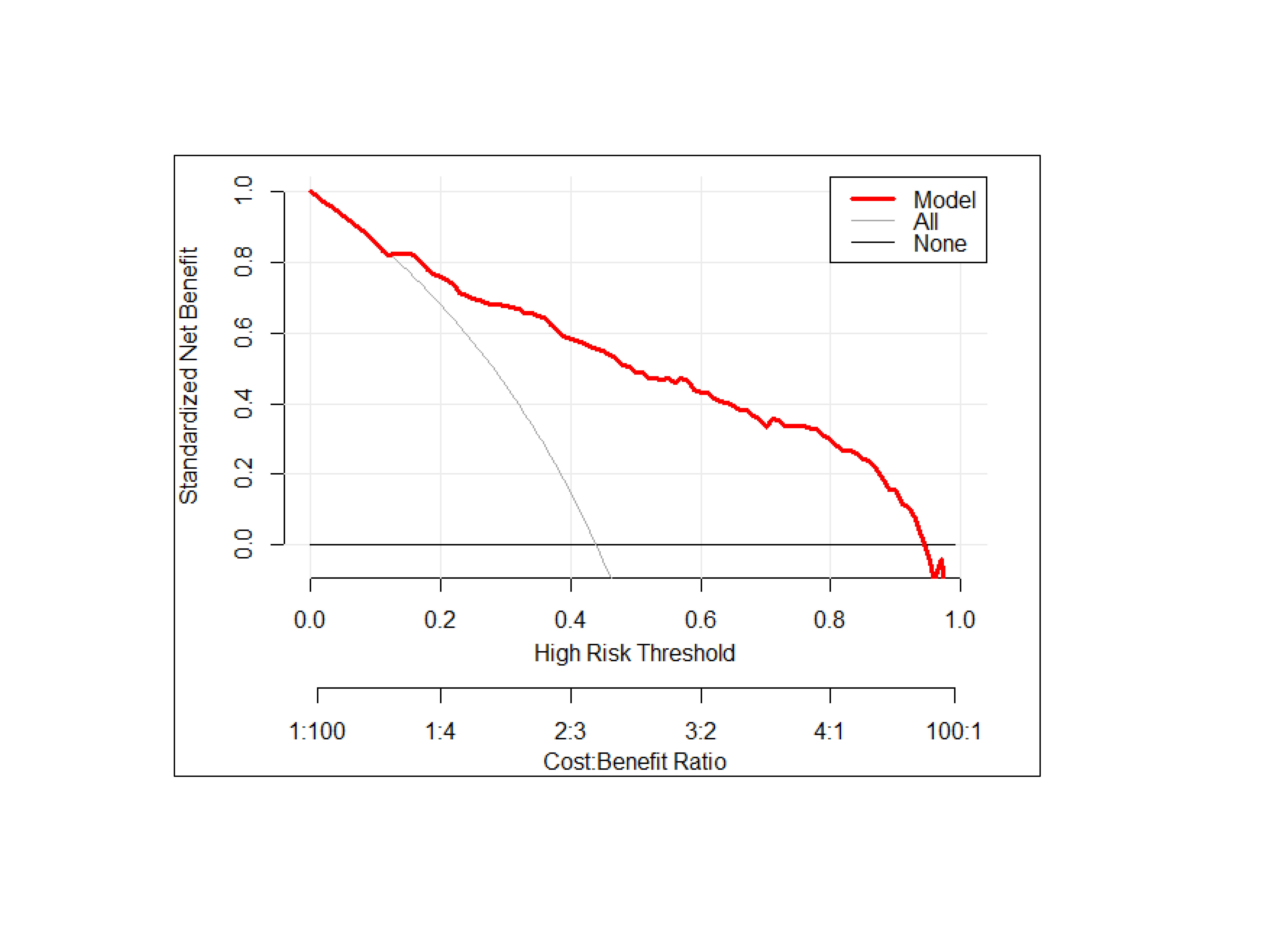

Supplement: Supplementary Figure 4 — Decision curve analysis was exhibited to estimate the clinical usefulness of the nomogram (csPCa). The quantified net benefits can be measured at different threshold probabilities. The y-axis denotes the standardized net benefit, and the x-axis denotes the threshold probabilities. The red line represents our nomogram, the gray line represents the condition that all patients have csPCa, and the black line represents the condition that none have csPCa. [file Image_4.tif]

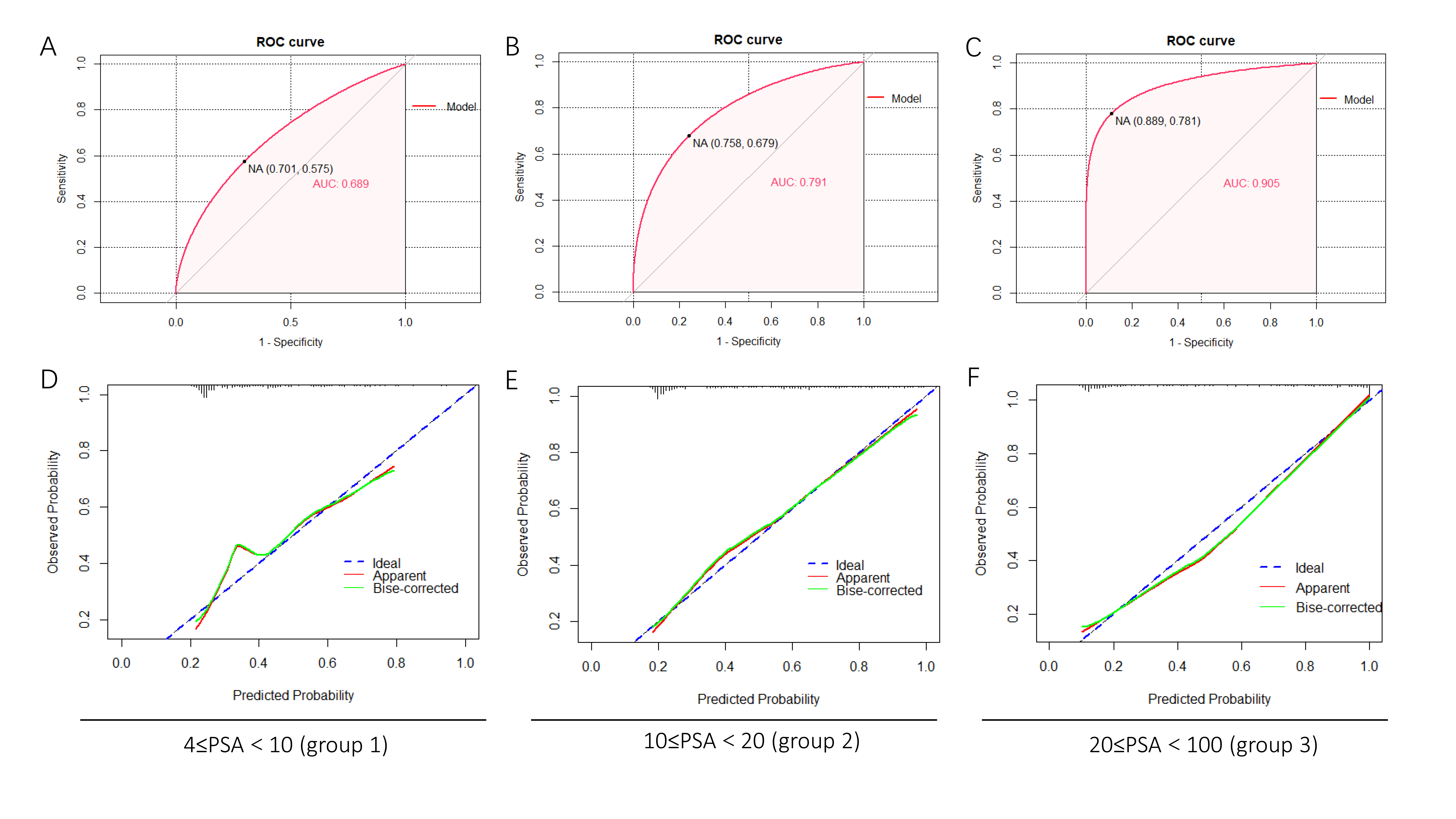

Supplement: Supplementary Figure 5 — Internal validation of the nomogram in the KD cohort for three PSA groups. (A, B, and C) ROC curve of the three groups for assessing the discrimination. (D, E, and F) Calibration plots of the three groups for assessing the calibration. [file Image_5.tif]

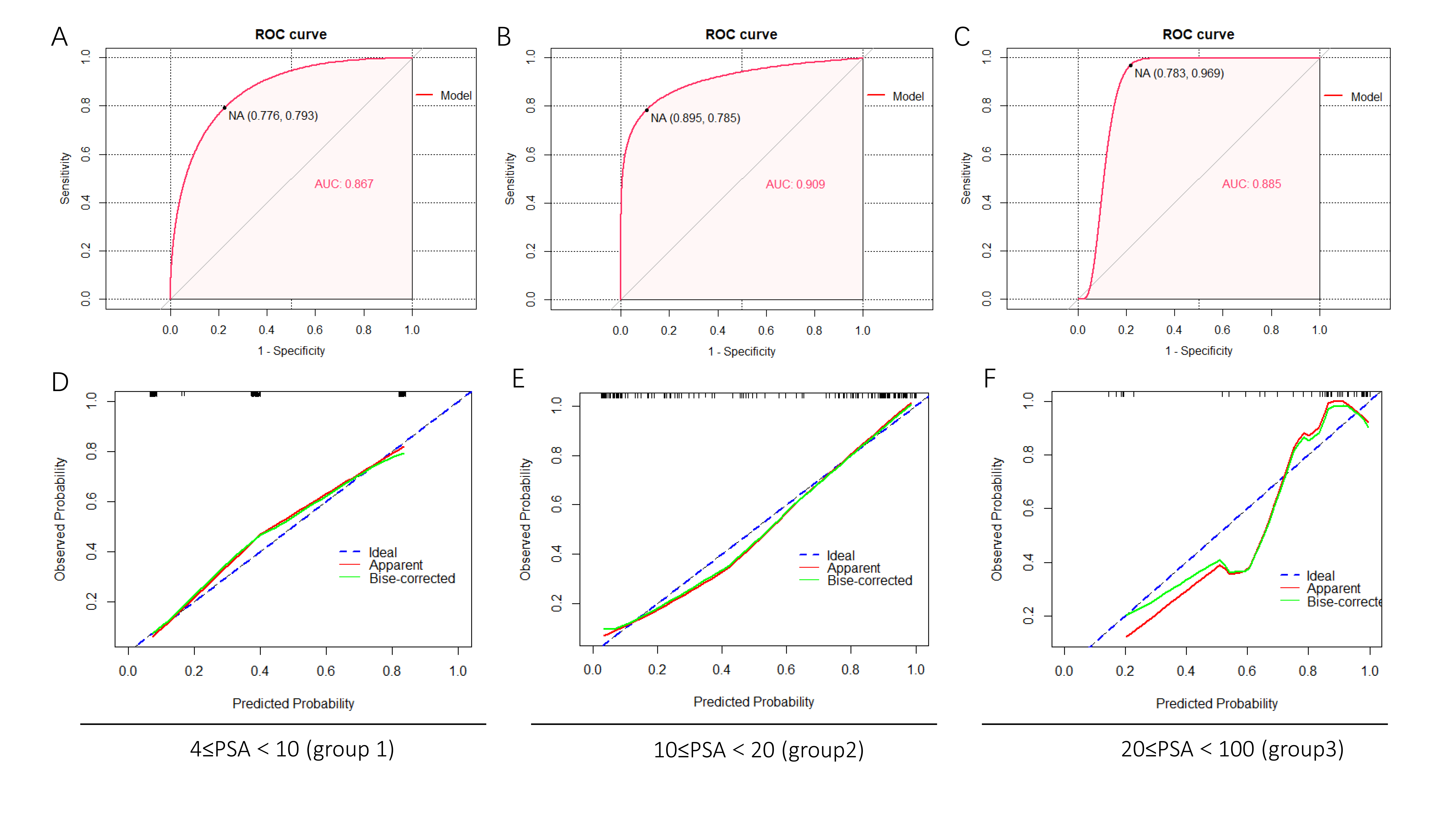

Supplement: Supplementary Figure 6 — External validation of the nomogram in the DF cohort for three PSA groups. (A, B, and C) The ROC curve of the three groups for assessing the discrimination. (D, E, and F) Calibration plots of the three groups for assessing the calibration. [file Image_6.tif]

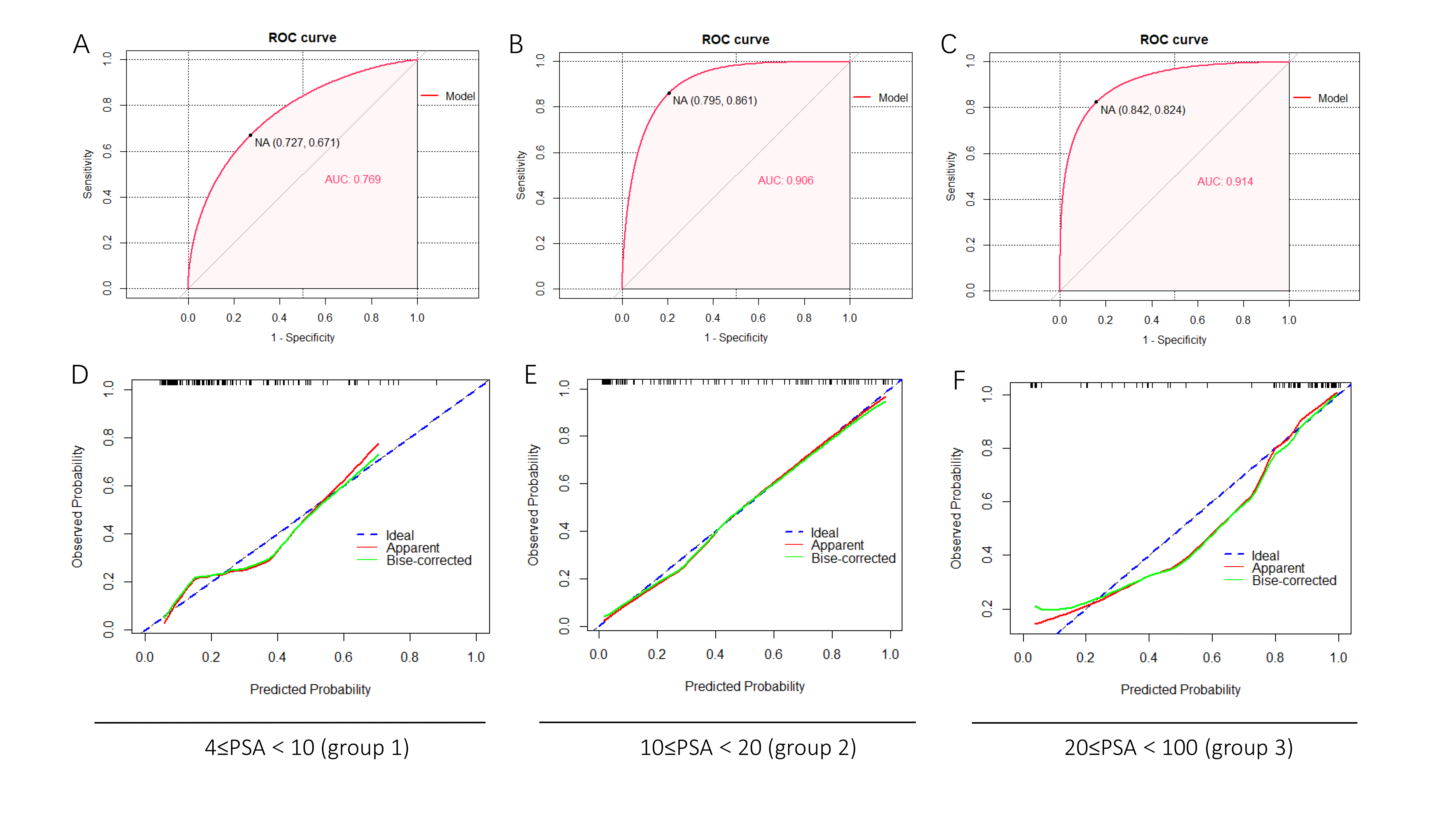

Supplement: Supplementary Figure 7 — External validation of the nomogram in the ZD cohort for three PSA groups. (A, B, and C) The ROC curve of the three groups for assessing the discrimination. (D, E, and F) Calibration plots of the three groups for assessing the calibration. [file Image_7.tif]
