# Supplementary material for: Construction and Validation of a Clinical Predictive Nomogram for Improving the Cancer Detection of Prostate Naive Biopsy Based on Chinese Multicenter Clinical Data
Source: Front Oncol. 2022 Jan 21;11:811866. doi: 10.3389/fonc.2021.811866 (PMC8814531; doi:10.3389/fonc.2021.811866)
Supplement: Supplementary Table 1 — Univariate and multivariate analysis for screening the predictors of outcomes (csPCa) of prostatic biopsy. csPCa, clinically significant prostate cancer; BMI, body mass index; PSA, prostate-specific antigen; PSAD, prostate-specific antigen density; PI-RADS, prostate imaging-reporting and data system; OR, odds ratio; CI, confidence interval. [file Table_1.docx]

| Parameters | Univariate model | | |  | Multivariate model | | | |
| --- | --- | --- | --- | --- | --- | --- | --- | --- |
|  | OR | 95%CI | P |  | B | OR | 95%CI | P |
| Age (years) | 1.079 | 1.056-1.101 | <0.001 |  |  |  |  |  |
| BMI (kg/m^2^) | 1.067 | 1.003-1.135 | 0.041 |  |  |  |  |  |
| PSA (ng/ml) | 1.018 | 1.010-1.027 | <0.001 |  |  |  |  |  |
| PSAD | 11.867 | 7.377-19.090 | <0.001 |  | 0.908 | 2.480 | 1.947-3.157 | <0.001 |
| PI-RADS grade | 4.083 | 3.301-5.051 | <0.001 |  | 1.562 | 4.769 | 3.013-7.548 | <0.001 |

**TABLE S1** Univariate and multivariate analysis for screening the predictors of outcomes (csPCa) of prostatic biopsy

*csPCa, clinically significant prostate cancer; BMI, body Mass Index; PSA, prostate-specific antigen; PSAD, prostate-specific antigen density; PI-RADS, prostate imaging-reporting and data system; OR, odds ratio; CI, confidence interval.*
